# Synthetic Frog-Derived-like Peptides: A New Weapon against Emerging and Potential Zoonotic Viruses

**DOI:** 10.3390/v15091804

**Published:** 2023-08-24

**Authors:** Annalisa Chianese, Valentina Iovane, Carla Zannella, Carla Capasso, Bianca Maria Nastri, Alessandra Monti, Nunzianna Doti, Serena Montagnaro, Ugo Pagnini, Giuseppe Iovane, Anna De Filippis, Massimiliano Galdiero

**Affiliations:** 1 Department of Experimental Medicine, University of Campania “Luigi Vanvitelli”, 80138 Naples, Italy; annalisa.chianese@unicampania.it (A.C.); carla.zannella@unicampania.it (C.Z.); carlacapasso98@hotmail.it (C.C.); biancamaria.nastri@unicampania.it (B.M.N.); anna.defilippis@unicampania.it (A.D.F.); 2 Department of Agricultural Sciences, University of Naples Federico II, 80055 Naples, Italy; valentina.iovane@unina.it; 3 Institute of Biostructures and Bioimaging (IBB), National Research Council (CNR), 80131 Naples, Italy; alessandra.monti@ibb.cnr.it (A.M.); nunzianna.doti@cnr.it (N.D.); 4 Department of Veterinary Medicine and Animal Productions, University of Naples “Federico II”, Via Delpino 1, 80137 Naples, Italy; serena.montagnaro@unina.it (S.M.); ugo.pagnini@unina.it (U.P.); giuseppe.iovane@unina.it (G.I.); 5 UOC of Virology and Microbiology, University Hospital of Campania “Luigi Vanvitelli”, 80138 Naples, Italy

**Keywords:** zoonotic infection, emerging viruses, AMPs, CDV, BVDV, SBV, CpHV-1, BoHV-1

## Abstract

Given the emergence of the coronavirus disease 2019 (COVID-19)**,** zoonoses have raised in the spotlight of the scientific community. Animals have a pivotal role not only for this infection, but also for many other recent emerging and re-emerging viral diseases, where they may represent both intermediate hosts and/or vectors for zoonoses diffusion. Today, roughly two-thirds of human infections are derived from animal origins; therefore, the search for new broad-spectrum antiviral molecules is mandatory to prevent, control and eradicate future epidemic outbreaks. Host defense peptides, derived from skin secretions of amphibians, appear as the right alternative to common antimicrobial drugs. They are cationic peptides with an amphipathic nature widely described as antibacterial agents, but less is reported about their antiviral potential. In the present study, we evaluated the activity of five amphibian peptides, namely RV-23, AR-23, Hylin-a1, Deserticolin-1 and Hylaseptin-P1, against a wide panel of enveloped animal viruses. A strong virucidal effect was observed for RV-23, AR-23 and Hylin-a1 against bovine and caprine herpesviruses, canine distemper virus, bovine viral diarrhea virus, and Schmallenberg virus. Our results identified these three peptides as potential antiviral-led candidates with a putative therapeutic effect against several animal viruses.

## 1. Introduction

Most emerging viral infectious diseases in humans are zoonotic, and able to spread from domestic or wild animals to humans via direct contact with blood, secretions, or other infected material, and through food, water, or environment. They can have devastating effects on the human population, causing significant economic losses and posing serious threats to public health [1,2]. Nipah virus, Ebola virus, Marburg virus, Lassa virus, severe acute respiratory syndrome, coronavirus (SARS-CoV and SARS-CoV-2), and Menangle virus are only some examples of viruses causing infectious diseases in recent decades. Animals serve as potential amplifier hosts, reservoirs or vectors of zoonotic infections [3,4,5]. In particular, livestock, such as poultry, sheep, and goats, are particularly harmful to humans, due to the close contact in which they live. However, the higher prevalence of emerging and re-emerging viruses, together with the lack of effective vaccines and antiviral therapies, have highlighted the need to develop new effective strategies against viruses resistant to available drugs. To prevent and control the spread of emerging zoonotic viral infectious diseases, it is important to follow the evolution and potential risks of various zoonotic viruses, in order to elucidate their pathogenesis and develop innovative antiviral strategies to limit their spread.

In this context, antimicrobial peptides (AMPs), or also called host defense peptides (HDPs), have aroused greater interest [6]. They derive from different sources, such as bacteria, plants, arthropods, reptiles, and insects [7,8]. Several AMPs derived from the skin secretion of amphibians colonize hostile habitats and expose their mucous surfaces to continuous and direct contact with microbes and other agents. In this regard, frogs release AMPs in the dermal glands through a holocrine mechanism to defend themselves [9,10]. According to the literature, there are many peptides secreted by different species of amphibians that, despite belonging to different families, share structural, chemical, and physical characteristics [11,12,13]. They are small cationic peptides of 10-50 amino acid residues with a net charge generally between +2 and +6. In addition, they tend to structure in amphipathic alpha-helix conformation [6,9]. Several frog AMPs were identified from 1985 to 2019 [14]. The first was secreted by the European frog and exhibited strong antibacterial activity against Gram-positive and Gram-negative bacteria [15]. Subsequently, other peptides were isolated from different amphibians, such as pseudin-2, secreted by the South American frog *Pseudis paradox* [16]. Olson et al. demonstrated that pseudin-2 could inhibit the *Echerichia coli* and *Staphylococcus aureus* infection with a minimum inhibitory concentration (MIC) at 2.5 µM and 80 µM, respectively. On the contrary, the peptide did not show a remarkable antifungal activity, with MIC at 130 µM against *Candida albicans* [16]. 

On the one hand, amphibian AMPs have been widely described for their antibacterial activity; on the other hand, their antiviral effect was less elucidated. Despite their unclear mechanism of action, these peptides could prevent viral infections through both extracellular and intracellular mechanisms. AMPs can prevent viral attack by binding to targets present on the host cell membrane and by competing for the binding site; they may interfere with viral particles by exerting a virucidal effect or blocking the fusion/entry of the virus into the host cell. They can also act at the intracellular level by inhibiting various stages of the viral cycle. However, multiple studies reported that most AMPs tend to act by changing the curvature of the viral membrane, thus reducing susceptibility to infection [9,17]. Beloid et al. demonstrated that dermaseptins derived from the *Phyllomedusa* genus exerted a virucidal effect against a broad spectrum of viruses, such as herpes simplex virus type 1 (HSV-1) and 2 (HSV-2), and human immunodeficiency virus type 1 (HIV-1) [18,19]. In addition, magainins 1 and 2, secreted by *Xenopus laevis*, exhibited a strong inhibitory activity against herpesviruses [20]. Additionally, peptides derived from the Australian tree frog, namely caerin 1.1 (secreted by *Litoria caerula)* and caerin 1.9 (isolated from *Litoria chlaris)*, had a strong virucidal effect against HIV-1 [21]. Another peptide resulting from the skin secretion of amphibians is urumin, secreted by *Hydrophylax bahuvistara*. As demonstrated by Agamennone et al, this peptide inhibited influenza virus infection by interacting with hemagglutinin [22]. Recently, we demonstrated that Temporin L, belonging to the Ranidae family and secreted by *Rana temporaria*, not only had antibacterial activity, but was also capable of inhibiting viral infection. Indeed, the peptide and its analogs showed a virucidal effect against herpesviruses (HSV-1, HSV-2), coronaviruses (SARS-CoV-2, HCoV-229E, HCoV-OC43), influenza virus and paramyxoviruses (measles virus and human parainfluenza virus type 3) [9].

Here, we analyzed the antiviral activity of several peptides secreted by different species of amphibians. We selected AR-23, derived from *Rana tagoi*, and RV-23, secreted by *Rana draytonii*, each consisting of 23 amino acid residues and characterized by an amphipathic α-helical structure and melittin-like sequences. Additionally, Hylin-a1, isolated from *Heleioporus albopunctatus,* Hylaseptin-P1, derived from *Leptopelis aubryi*, and Deserticolin-1, secreted by *Crinia deserticola*, were analyzed via plaque assays for their inhibitory effect against canine distemper virus (CDV, *Paramyxoviridae*), bovine viral diahrrea virus (BVDV, *Flaviviridae*), Schmallenberg virus (SBV, *Bunyaviridae*), and animal herpesviruses, namely bovine herpesvirus type 1 (BoHV-1) and caprine herpesvirus type 1 (CpHV-1). 

## 2. Materials and Methods

### 2.1. Chemistry

#### Peptide Synthesis and Characterization

Protected amino acids, coupling agents (HATU, Oxyma) and Fmoc-Rink Amide AM resin used for peptide synthesis, solvents, including acetonitrile (CH_3_CN), dimethyl-formamide (DMF) and other products such as trifluoroacetic acid (TFA), symcollidine, diisopropylethylamine (DIPEA), and piperidine, were purchased from Merck (Milan, Italy). Peptides AR-23 (single letter sequence: H-AIGSILGALAKGLPTLISWIKNR-NH2), RV-23 (single letter sequence: H-RIGVLLARLPKLFSLFKLMGKKV-NH2), Hylin-a1 (single letter sequence: H-IFGAILPLALGALKNLIK-NH2), Deserticolin-1 (single letter sequence: H-GLADFLNKAVGKVVDFVKS-NH2) and Hylaseptin-P1 (single letter sequence: H-GILDAIKAIAKAAG-OH), were synthesized using oxyma/DIC as coupling agents and following methods reported in the literature [6,23]. High-Performance Liquid Chromatography (HPLC preparative purification was carried out on a WATERS 2545 preparative system (Waters, Milan, Italy) fitted out with a WATERS 2489 UV/Visible detector, applying a linear gradient of CH_3_CN/0.05%TFA in water 0.05% TFA from 5 to 70% in 20 min, at a flow rate of 12 mL/min. Mass spectrometry (MS) characterization of peptides was performed using an ESI-TOF-MS Agilent 1290 Infinity LC System coupled to an Agilent 6230 time-of-flight (TOF) LC/MS System (Agilent Technologies, Cernusco sul Naviglio, Italy). The LC Agilent 1290 LC module was coupled with a photodiode array (PDA) detector and a 6230 time-of-flight MS detector, along with a binary solvent pump degasser, a column heater, and an autosampler. Liquid chromatography (LC)-MS characterization of peptides was performed using a C18 Waters xBridge column (3 μm, 4.6 × 5.0 mm), applying a linear gradient of CH_3_CN/0.05% TFA in water 0.05% TFA from 5 to 70% in 20 min, at a flow rate of 0.2 mL/min. The LC–MS analysis of pure peptides is reported in the Supplementary Figure S1. The yields of target peptides, calculated as ((experimental weight of pure peptide)/(theoretical weight) × 100), where the theoretical weight was calculated based on the synthesis scale used, were estimated to be about 70%. The relative purity of peptides was calculated as the ratio of peak area of the target peptide and the sum of areas of all detected peaks from the UV chromatograms at 210.4 nm. The purity was up to 95%.

### 2.2. Biology

#### 2.2.1. Cell Culture and Viral Strain

Vero/hSLAM cells (ECACC 04091501, Porton Down, United Kingdom), Baby hamster kidney cells (BHK-21, ATCC CCL-10, Manassas, VA, USA) and Madin Darby bovine kidney cells (MDBK, ATCC CCL-22,) were grown in Dulbecco’s Modified Eagle Medium (DMEM) with 4.5 g/L glucose (Microtech, Naples, Italy) supplemented with 100 IU/mL penicillin and 100 μg/mL streptomycin (antibiotic solution 100×; Himedia, Naples, Italy) and 10% fetal bovine serum (Microtech, Naples, Italy).

CDV (strain Onderstepoort) was propagated on VERO/hSLAM cells, while BoHV-1 (Cooper strain), CpHV-1 (reference Swiss strain E/CH), and BVDV (strain NADL ATCC VR-534) were grown on MDBK cell line. Finally, SBV was propagated on BHK-21 monolayers. 

All cell lines were infected with 0.01 multiplicity of infection (MOI), and the viral titers were evaluated by plaque assay. The original stock titers of the viruses used in this study were 10^7^ plaque-forming units (PFU)/mL for CDV, BoHV-1, CpHV-1 and BVDV. SBV was titrated via tissue culture infective dose (TCID50) by the Reed and Muench endpoint titration method. 

#### 2.2.2. Cytotoxic Activity 

Vero/hSLAM, BHK-21, and MDBK cells were seeded in 96-well microtiter tissue culture plates and incubated 24 h at 37 °C in 5% CO_2_. Subsequently, cells were incubated with two-fold dilutions of peptides (from 0.39 to 100 μM) for 2 and 24 h. The cytotoxicity was evaluated via the 3-(4,5-dimethylthiazol-2-yl)-2,5-diphenyl-2H-tetrazolium bromide (MTT) [9] by measuring the absorbance at 570 nm. Negative control (ctr-) was represented by cells treated with DMSO 100%, while positive control (ctr+) were non-treated cells. All experiments were performed in triplicate, and the means standard deviations were reported. Nonlinear regression analysis was performed using GraphPad Prism software (GraphPad Software, version 8.0.1) to determine the 50% cytotoxic concentration (CC_50_).

#### 2.2.3. Antiviral Activity

The antiviral activity of peptides against CDV, CpHV-1, and BoHV-1 was evaluated through four different assays, in which the main difference is the timing of the addition of peptides [9].

Vero/hSLAM and MDBK cells were seeded in 24-well plates (1.2 × 10^5^ cells) 24 h before use. In co-treatment assay, cells were inoculated simultaneously with noncytotoxic concentrations of peptides and virus at MOI 0.01 for 1 h at 37 °C/5% CO_2_. In virus pre-treatment assay, peptides were incubated with the virus at MOI 0.1 for 1 h at 37 °C. After that, the mixture was diluted and inoculated on the cells for another hour at 37 °C/5% CO_2_. In cell pre-treatment assay, cells were first pre-treated with peptides and then infected with the virus (MOI 0.01) at 37 °C/5% CO_2_. Lastly, in post-treatment assay, cells were infected with the virus (MOI 0.01) for 1 h, and subsequently, peptides were added for another hour at 37 °C/5% CO_2._ At the end of the viral adsorption time, for all the assays above mentioned, complete medium supplemented with carboxymethylcellulose (CMC) 5% (Sigma, C5678, C5013) was added and incubated for 72 h at 37 °C in 5% CO_2_. The cells were fixed with 4% formaldehyde (Sigma, F1635), stained with crystal violet 0.5%, and the number of plaques scored. The percentage of viral inhibition was calculated compared to the infected control (ctr-) as follows:

% viral inhibition = (100 − (plaques counted in the test sample)/plaques estimated in the negative control)) × 100.

The antiviral activity of peptides against SBV was performed by TCID50 assay. BHK-21 were seeded in 96-well microtiter tissue culture plates (2 × 10^4^ cells) and incubated for 24 h. The next day, several concentrations of peptides and SBV were incubated for 1 h at 37 °C/5% CO_2_. Then, 100 µL of the diluted mixture was inoculated on the cell monolayer, wells were monitored for a cytopathic effect (CPE) for 72 h, and the Reed and Munch method was used to calculate the results as 10× TCID50 mL.

### 2.3. Statistical Analysis

All tests were performed in triplicate and expressed as mean ± standard deviation (SD) calculated by GraphPad Prism (version 8.0.1). One-way ANOVA followed Dunnett’s multiple comparisons test was performed; a value of *p*  ≤ 0.05 was considered significant.

## 3. Results

### 3.1. Evaluation of Cytotoxicity on Cellular Models

**Figure 1:**
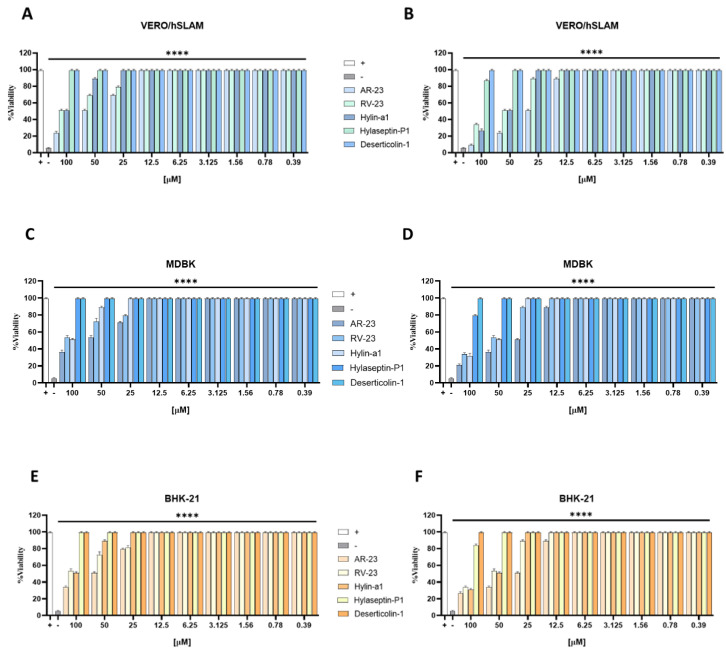
Toxicity analysis. Peptide toxicity was evaluated by MTT assay after 2 h (**A**,**C**,**E**) and 24 h (**B**,**D**,**F**) of peptide treatment on (**A**,**B**) VERO/hSLAM cells, (**C**,**D**) MDBK cells, and (**E**,**F**) BHK-21 cells. **** *p* < 0.0001; ns: non-significant.

The effects of peptides on cell viability were examined after incubating VERO/hSLAM, BHK-21, and MDBK cell lines with different concentrations of each peptide for 2 and 24 h. Cytotoxicity was investigated by an MTT assay and expressed as a percentage of viability compared to the untreated control cells. The peptides exhibited a dose-dependent cytotoxicity, as illustrated in Figure 1. After 2 h incubation, RV-23 and Hylin-a1 showed the lower toxicity towards all cellular models, with a very similar 50% cytotoxic concetration (CC_50_) at 100 µM; whereas AR-23 exhibited a CC_50_ at 50 μM in the same experimental conditions. On the contrary, after 24 h, the nonlinear regression analysis data indicated that CC_50_ was 25 μM for AR-23 and 50 µM for RV-23 and Hylin-a1 (Figure 1). The other two peptides, i.e., Deserticolin-1 and Hylaseptin-P1, were not cytotoxic even at higher concentrations tested.

The effects of peptides on cell viability were examined after incubating VERO/hSLAM, BHK-21, and MDBK cell lines with different concentrations of each peptide for 2 and 24 h. Cytotoxicity was investigated by an MTT assay and expressed as a percentage of viability compared to the untreated control cells. The peptides exhibited a dose-dependent cytotoxicity, as illustrated in Figure 1. After 2 h incubation, RV-23 and Hylin-a1 showed the lower toxicity towards all cellular models, with a very similar 50% cytotoxic concetration (CC_50_) at 100 µM; whereas AR-23 exhibited a CC_50_ at 50 μM in the same experimental conditions. On the contrary, after 24 h, the nonlinear regression analysis data indicated that CC_50_ was 25 μM for AR-23 and 50 µM for RV-23 and Hylin-a1 (Figure 1). The other two peptides, i.e., Deserticolin-1 and Hylaseptin-P1, were not cytotoxic even at higher concentrations tested.

### 3.2. Antiviral Activity 

#### 3.2.1. AR-23, RV-23 and Hylin-a1 Effect against CDV Infection

In order to understand the mechanism of action of peptides and to evaluate which step of the viral lifecycle they could interfere with, four different time of addition assays were performed, as described in the Section 2.

**Figure 2:**
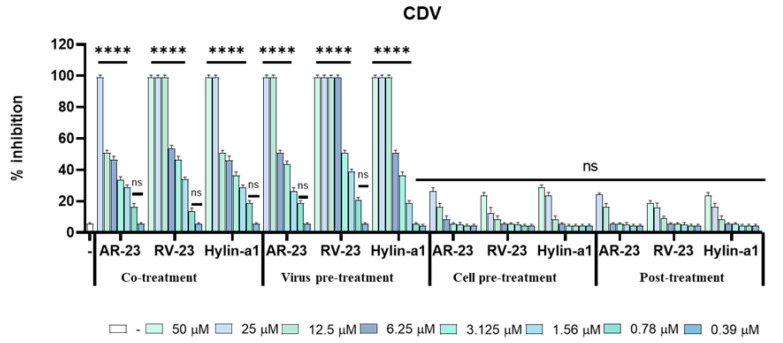
Antiviral activity against CDV. Different assays were performed to evaluate anti-CDV activity. From left to right: Co-treatment: simultaneous addition of peptide and virus to the cells; Virus pre-treatment: virus incubated with AR-23, RV-23 or Hylin-a1 and then used to infect cells; Cell pre-treatment: AR-23, RV-23 and Hylin-a1 incubated with the cells before the viral infection; Post-treatment: AR-23, RV-23 and Hylin-a1 added to the infected cells. Infected cells were used as negative control (ctr-). **** *p* < 0.0001; ns: non-significant.

The inhibitory effect against CDV infection was evaluated on VERO/hSLAM cells at not cytotoxic concentrations in the range from 25 to 0.39 µM for AR-23, and from 50 to 0.39 µM for RV-23 and Hylin-a1 (Figure 2). Peptides reduced viral titer in two treatment assays (co-treatment and virus pre-treatment), suggesting they could act in an early phase of the viral infection. 

The half-maximal inhibitory concentration (IC_50_) was determined. The values of IC_50_ for RV-23 corresponded to 6.25 µM when peptide and virus were inoculated on the VERO/hSLAM cells simultaneously (co-treatment), and 3.125 µM when peptides and virus were first pre-incubated together and after added on cells (virus pre-treatment) (Figure 2). AR-23 and Hylin-a1 similarly inhibited virus replication at higher concentrations than RV-23, showing an IC_50_ at 12.5 µM and 6.25 µM in co-treatment and virus-pre-treatment assay, respectively. On the contrary, any reduction of CDV infection was observed in the other two assays (cell-pre-treatment and post-treatment), suggesting that any peptides could interfere with cell surface receptors or viral replication inside the cell. Finally, Deserticolin-1 and Hylaseptin-P1 did not show an inhibitory effect in all the time-addition assays (Supplementary Figure S2).

#### 3.2.2. AR-23, RV-23 and Hylin-a1 Effect against SBV and BVDV Infection

The same in vitro treatments were performed to understand if peptides could inhibit viral replication of BVDV and SBV. In all the tested conditions, we observed an inhibitory activity trend similar to that reported for CDV, but with lower intensity.

**Figure 3:**
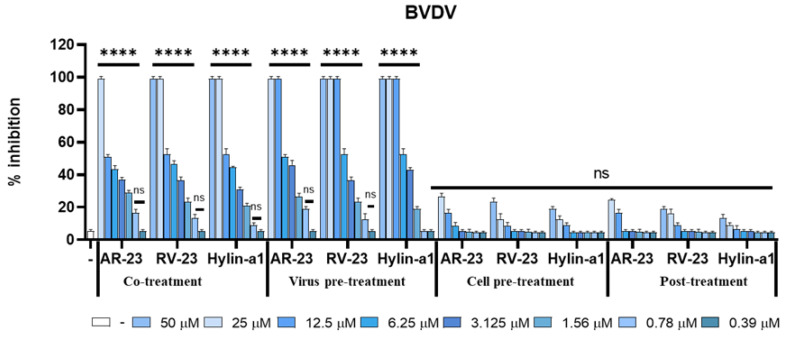
Antiviral activity against BVDV. Different assays were performed to evaluate anti-BVDV activity. From left to right: Co-treatment: simultaneous addition of peptides and virus to the cells; Virus pre-treatment: virus incubated with AR-23, RV-23, and Hylin-a1 and then used to infect cells; Cell pre-treatment: AR-23, RV-23, and Hylin-a13 incubated with the cells before the viral infection; Post-treatment: AR-23, RV-23, and Hylin-a1 added to the infected cells. Infected cells were used as negative control (ctr-). **** *p* < 0.0001; ns: non-significant.

Indeed, as reported in Figure 3, AR-23, RV-23, and Hylin-a1 showed the same inhibitory activity against BVDV with an IC_50_ at 12.5 and 6.25 µM in co-treatment and virus pre-treatment assays, respectively.

**Figure 4:**
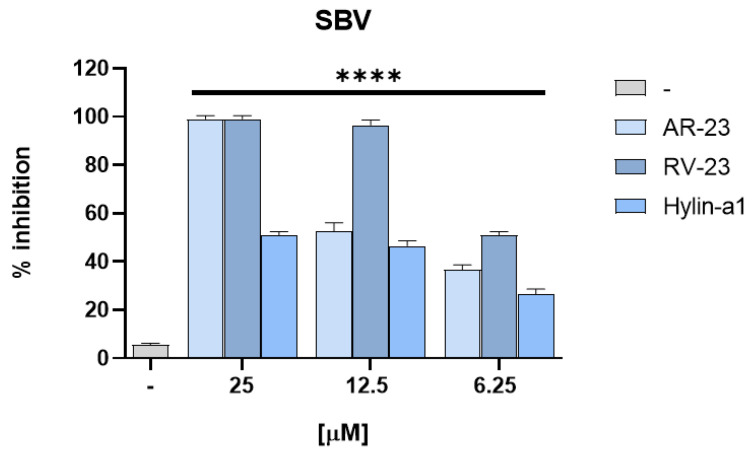
SBV (200 TCID50) was incubated with AR-23, RV-23 and Hylin-a1 at no cytotoxic concentrations (from 25 to 6.25 μM). After 1 h of incubation, the mixture was titrated on BHK-21 cells. CPE was detected 72 h after the infection, and cell viability was determined using MTT staining. **** *p* < 0.0001.

Furthermore, the inhibitory effect of peptides on SBV infection was also analyzed by performing TCID50 assay. The BHK-21 cell line was infected with the virus and treated with peptides at no cytotoxic concentrations (Figure 4). The CPE was monitored, and data were analyzed through a colorimetric assay. In Figure 4, we reported data obtained via MTT staining by reading the absorbance at spectrophotometer. The percentage of inhibition was related to the concentration of the tested peptides, confirming that RV-23 was able to reduce SBV infection with a higher efficacy (Figure 4). Additionally, in this case, Deserticolin-1 and Hylaseptin-P1 had any activity against BDVD and SBV (Supplementary Figure S3).

#### 3.2.3. AR-23, RV-23 and Hylin-a1 Effect against CpHV-1 and BoHV-1

**Figure 5:**
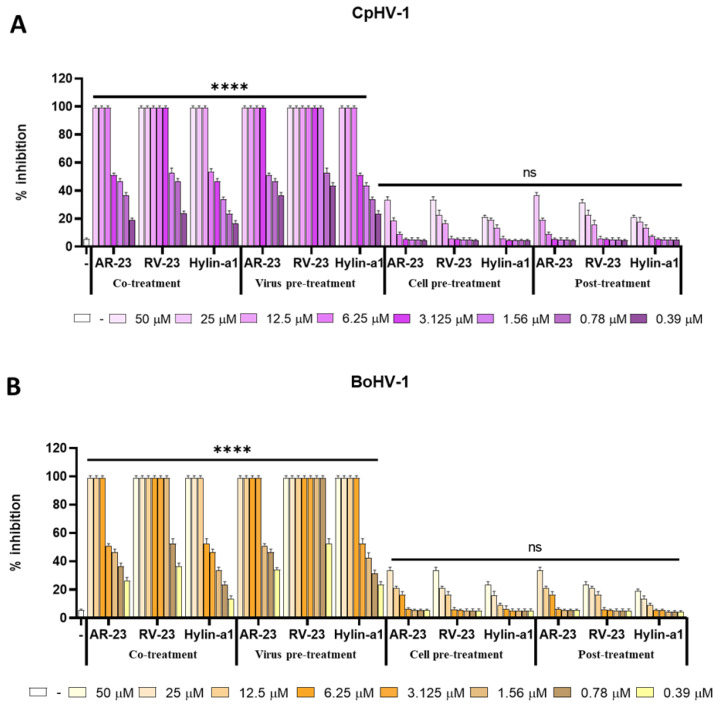
Antiviral activity against CpHV-1 and BoHV-1. Different assays were performed to evaluate anti-CpHV-1 (**A**) and anti-BoHV-1 (**B**) activity. From left to right: Co-treatment: simultaneous addition of peptides and virus to the cells; Virus pre-treatment: virus incubated with AR-23, RV-23, and Hylin-a1 and then used to infect cells; Cell pre-treatment: AR-23, RV-23 and Hylin-a1 incubated with the cells before the viral infection; Post-treatment: AR-23, Rv-23 and Hylin-a1 added to the infected cells. Infected cells were used as negative control (ctr-). **** *p* < 0.0001; ns: non-significant.

Finally, antiviral activity of peptides was investigated against CpHV-1 and BoHV-1, which are the main pathogens causing herpetic diseases in animals. The same time of addition assays were performed to investigate the virucidal effect of peptides. As reported in Figure 5, the three peptides showed a strong activity against CpHV-1 and BoHV-1 until very low concentrations. They inhibited the infection in co-treatment and virus pre-treatment assays confirming that the peptides were able to play a crucial role in early stage of viral lifecycle. RV-23 reduced the viral replication, especially when it was pre-treated with virus (virus pre-treatment) with an IC_50_ at 0.78 and 0.39 µM against CpHV-1 and BoHV-1, respectively (Figure 5A,B). In addition, AR-23 also exhibited a strong virucidal activity with IC_50_ at 1.56 µM for both the viruses (Figure 5A,B). Finally, Hylin-a1 inhibited the infection of both viruses at higher concentrations than AR-23 and RV-23 (IC50 at 3.125 µM in virus pre-treatment). As previously observed, Deserticolin-1 and Hylaseptin-P1 exerted no antiviral action against both viruses (Supplementary Figure S4).

## 4. Discussion

In recent years, several viral zoonoses pathogenic for humans occurred, while many other viruses are still endemic in several parts of the world. The large prevalence of viruses is due to the close contact between humans and domestic or wild animal reservoirs, and to the high diffusion of insect and arthropod vectors. Paramyxoviruses, bunyaviruses, and flaviviruses were reported among the main recent viruses able to cause zoonotic infections [24,25,26,27]. For instance, Nipah virus and Hendra virus are transmitted via flying foxes and, through an intermediate host (swine or equine), both viruses are able to infect humans and livestock and cause very severe outbreaks, sometimes deadly [28,29]. Other paramyxoviruses infect animals, such as CDV, that is closely related to the human measle virus. It is the causative agent of canine distemper (CD), an acute infectious disease primarily affecting dogs and other carnivores. Duo to its high cross-species transmission potential and to the probability to spill from animals to humans [30], CDV represents a global concern. Ruminant livestock is also considered an optimal reservoir of infectious diseases. Several viruses are confined in ruminants, such as BVDV, SBV, and different animal herpesviruses, but they possess a strong potential to jump into humans. Therefore, the establishment of early warning strategies and the search for new therapeutic approaches are needed in order to prevent, control and eradicate these infections that could critically impact our social, economic and health systems. 

Notably, it is estimated that most of the recent viral outbreaks have been caused by enveloped viruses [31]. Therefore, inhibitors of viral entry, able to act on the viral surface or by interfering with the virus-cell interaction, could be essential to prevent early the infection. In this study, we evaluated the antiviral activity of frog-derived peptides, namely AR-23, RV-23, Hylin-a1, Deserticolin-1 and Hylaseptin-P1, against a broad spectrum of animal viruses. 

The peptides AR-23 and RV-23 are widely described for their well-known antimicrobial activities. In detail, the peptides reported significant bactericidal activity against Gram-positive (*Staphylococcus aureus*, *Staphylococcus epidermidis*, and *Bacillus subtilis*) and Gram-negative bacteria (*Escherichia coli*, *Pseudomonas aeruginosa*, and *Klebsiella pneumoniae*) [32,33,34]. In addition, we have recently demonstrated that AR-23 was able to interfere with a broad spectrum of viruses by blocking viral infection in the early stages. It exhibited an inhibitory effect against herpesvirus (HSV-1), paramyxoviruses (MeV and HPIV-2), and coronaviruses (HCoV-229E and SARS-CoV-2) [6]. On the contrary, the antiviral potency of RV-23, Hylin-a1, Deserticolin-1 and Hylaseptin-P1 was completely unknown, while their antibacterial activity was widely described [13,35,36,37,38,39,40]. For instance, recently, we demonstrated that Hylin-a1 exerted a bactericidal action against *Staphylococcus aureus* multi-resistant strains [41]. 

We demonstrated that RV-23, AR-23 and Hylin-a1 were active in inhibiting the infection of all the enveloped animal viruses used in the present study. All the peptides shared a common mechanism of inhibition by interacting directly with the viral particle in the early phase of infection. In detail, RV-23 exhibited the best activity with an IC_50_ ranging from 3 μM to 6 μM for CDV, SBV and BVDV, respectively (virus pre-treatment, Figure 2, Figure 3 and Figure 4). The antiviral effect was significantly increased against animal herpesviruses, where RV-23 inhibited the infection until very low micromolar concentrations (0.39 μM against BoHV-1, and 0.78 μM against CpHV-1, as indicated in the virus pre-treatment assay (Figure 5). The antiviral activity resulted lower when the virus was pre-treated with AR-23 and Hylin-a1 (Figure 2, Figure 3 and Figure 4), while Deserticolin-1 and Hylaseptin-P1 showed any effect (Supplementary Figures S2–S4). We hypothesized that the diverse behavior of peptides could be explained by their different positive charge, in the order RV-23 (+7) > AR-23 (+4) > Hylin-a1 (+3) > Deserticolin-1 (+2) > Hylaseptin-P1 (+1). The antiviral effect of peptides was due to the interaction with the negative-charged viral membrane, which probably caused a physical disruption of the lipid bilayer of the viral envelope (virolysis). Then, the higher cationic character of RV-23 could justify its greater antiviral activity since the peptide, due to interfacial hydrophobic and/or amphipathic propensity, could inhibit the viral infection by direct contact with the hydrophobic envelope.

## 5. Conclusions

New antiviral drugs need to be discovered and validated to overcome future epidemics caused by emerging and re-emerging viruses; moreover, most of the enveloped viruses are developing an increased resistance to antiviral drugs. In this scenario, entry inhibitors are a promising but unexplored target in the search for new antiviral alternatives. Our findings demonstrated that three frog-derived peptides, namely RV-23, AR-23 and Hy-lin-a1, interfered with the infection of CDV, BVDV, SBV, BoHV-1 and CpHV-1, acting as entry inhibitors, probably through a physical interaction with the hydrophobic viral surfaces. Further studies and a better characterization with in vitro and in vivo assays will be needed to proceed with their clinical application in humans.

## Data Availability

The data presented in this study are available on request from the corresponding author. Authors can confirm that all relevant data are included in the article.

## Supplementary Materials

The following supporting information can be downloaded at: https://www.mdpi.com/article/10.3390/v15091804/s1, Figure S1: TIC (Total Ion Chromatogram) profile and ESI-MS (ElectroSpray Ionization-Mass Spectrometry) spectra of purified antimicrobial peptides tested in this study; Figure S2: Antiviral activity against CDV; Figure S3: Antiviral activity against BVDV; Figure S4: Antiviral activity against CpHV-1 and BoHV-1.
