# Supplementary material for: Synthetic Frog-Derived-like Peptides: A New Weapon against Emerging and Potential Zoonotic Viruses
Source: Viruses. 2023 Aug 24;15(9):1804. doi: 10.3390/v15091804 (PMC10537403; doi:10.3390/v15091804)
Supplement: Supplementary file 1 [file viruses-15-01804-s001.zip › viruses-2577536-supplementary.pdf]

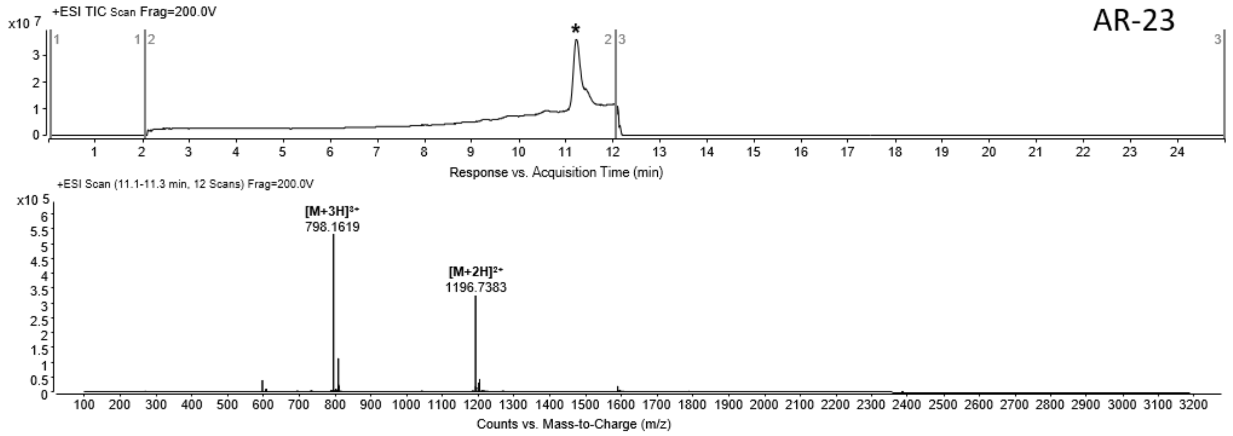
AR-23
+ESI TIC Scan Frag=200.0V
Response vs. Acquisition Time (min)
+ESI Scan (11.1-11.3 min, 12 Scans) Frag=200.0V
[M+3H]³⁺
798.1619
[M+2H]²⁺
1196.7383
Counts vs. Mass-to-Charge (m/z)

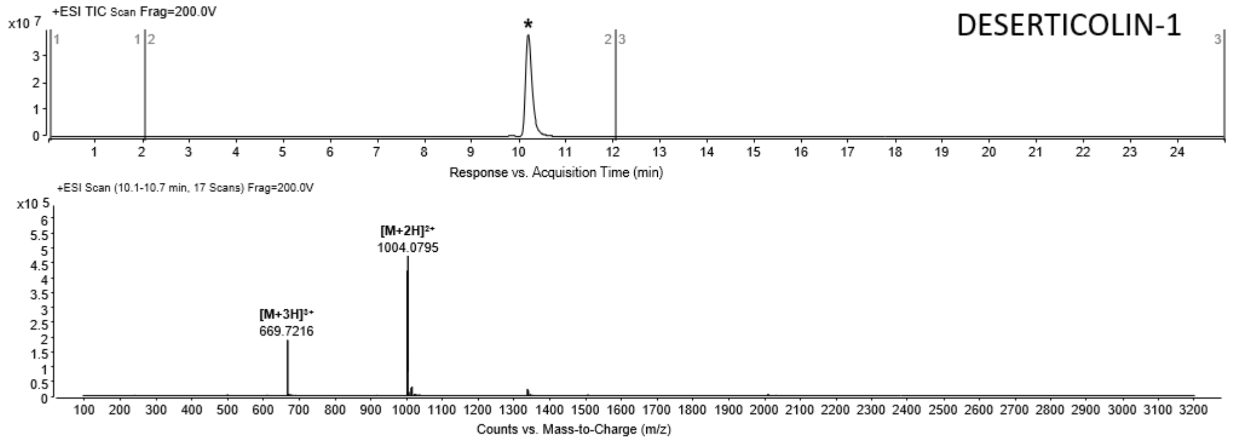
DESERTICOLIN-1
+ESI TIC Scan Frag=200.0V
Response vs. Acquisition Time (min)
+ESI Scan (10.1-10.7 min, 17 Scans) Frag=200.0V
[M+2H]²⁺
1004.0795
[M+3H]³⁺
669.7216
Counts vs. Mass-to-Charge (m/z)

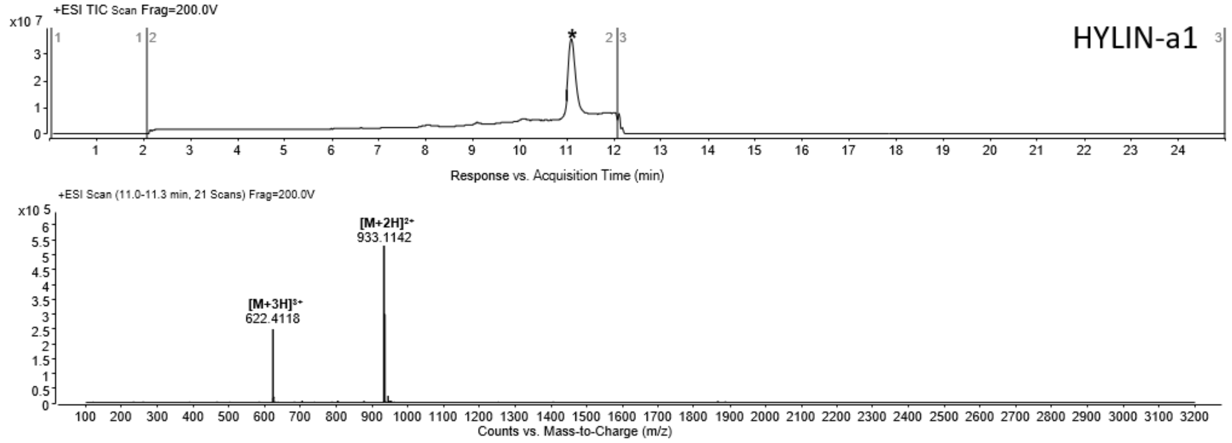
HYLIN-a1
+ESI TIC Scan Frag=200.0V
Response vs. Acquisition Time (min)
+ESI Scan (11.0-11.3 min, 21 Scans) Frag=200.0V
[M+2H]²⁺
933.1142
[M+3H]³⁺
622.4118
Counts vs. Mass-to-Charge (m/z)

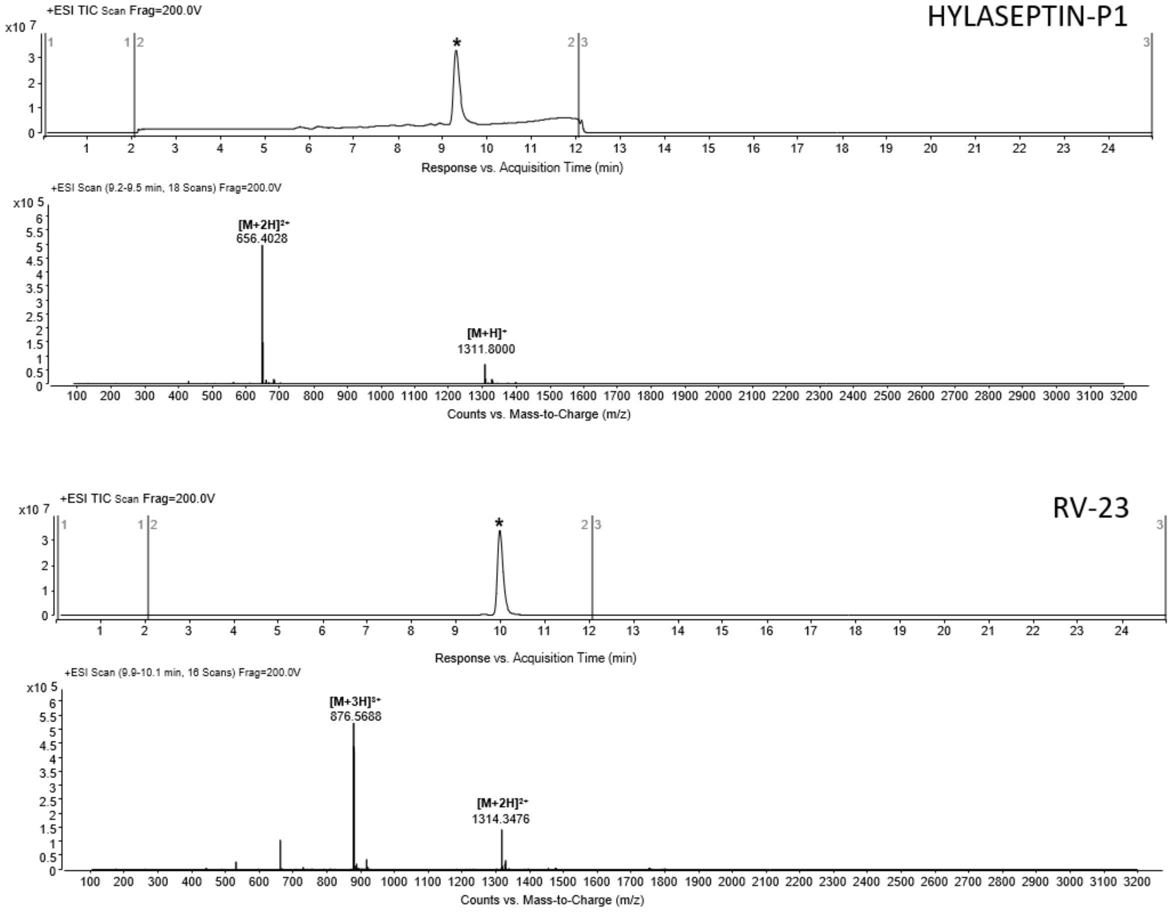

Figure S1. TIC (Total Ion Chromatogram) profile and ESI-MS (ElectroSpray Ionization-Mass Spectrometry) spectra of purified antimicrobial peptides tested in this study. In all the analyses we detected the expected peak of intact peptides at the retention time (tR) value of about 11.2 min for AR-23**,** 10.2 min for Deserticolin-1, 11.1 min for Hylin-a1, 9.3 min for Hylaseptin-P1 and 10.0 min for RV-23. The asterisk (*) indicates the chromatographic peak containing the target peptide (upper panels). ESI-MS analysis (lower panels) showed the expected mass at m/z: 1196.738 ($[M+2H]^{2+}$) and 798.162 ($[M+3H]^{3+}$) for AR-23; at m/z: 1004.079 ($[M+2H]^{2+}$) and 669.722 ($[M+3H]^{3+}$) for Deserticolin-1; at m/z: 933.114 ($[M+2H]^{2+}$), 622.411 ($[M+3H]^{3+}$) for Hylin-a1; at m/z: 1311.800 ($[M+H]^{+}$) and 656.403 ($[M+2H]^{2+}$) for Hylaseptin-P1; at m/z: 1314.348 ($[M+2H]^{2+}$), 876.569 ($[M+3H]^{3+}$) for RV-23.

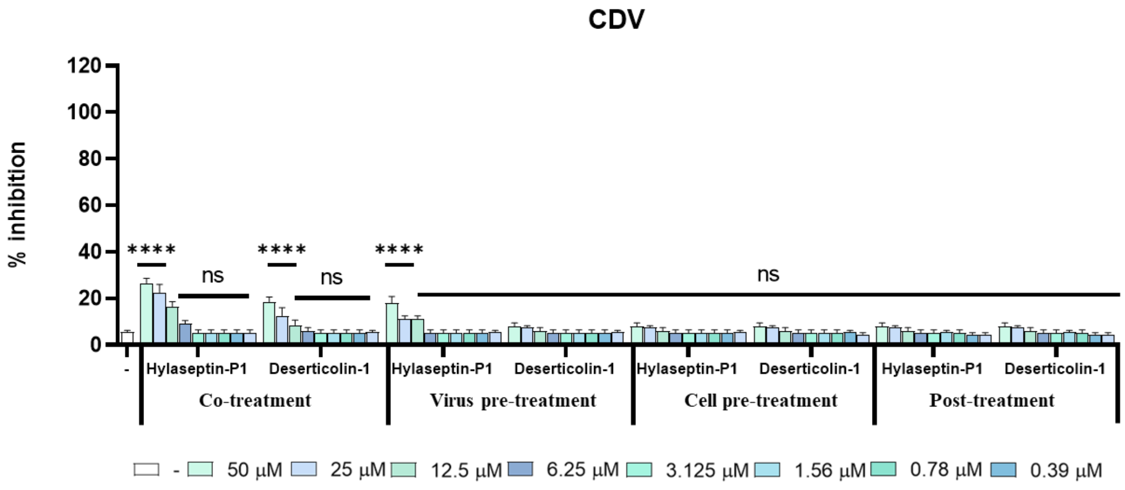

Figure S2. Antiviral activity against CDV. Different assays were performed to evaluate anti-CDV activity. From left to right: Co-treatment: simultaneous addition of peptide and virus to the cells; Virus pre-treatment: virus incubated with Hylaseptin-P1 and Deserticolin-1 and then used to infect cells; Cell pre-treatment: Hylaseptin-P1 and Deserticolin-1 incubated with the cells before the viral infection; Post-treatment: Hylaseptin-P1 and Deserticolin-1 added to the infected cells. Infected cells were used as negative control (ctr-). **** $p < 0.0001$; ns: non-significant.

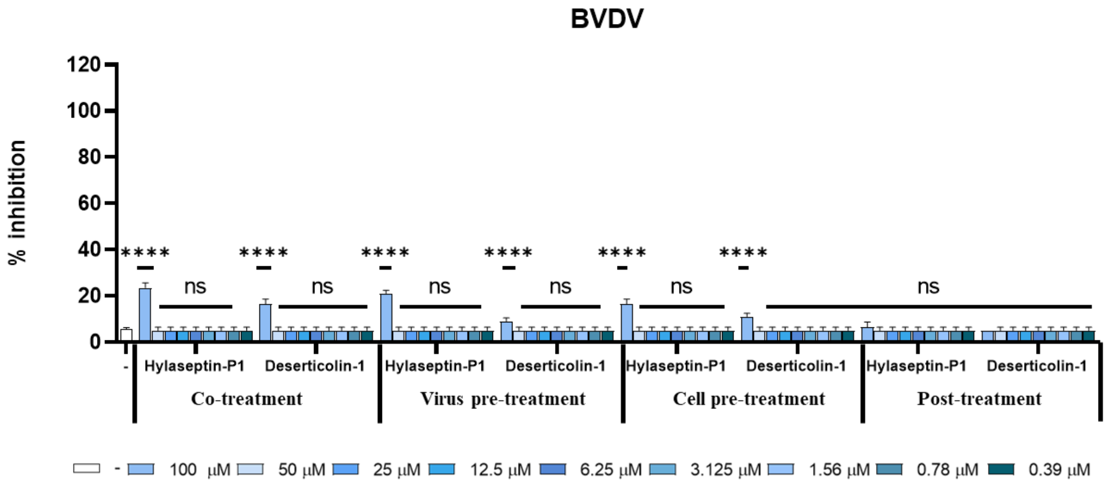

Figure S3. Antiviral activity against BVDV. Different assays were performed to evaluate anti-BVDV activity. From left to right: Co-treatment: simultaneous addition of peptide and virus to the cells; Virus pre-treatment: virus incubated with Hylaseptin-P1 and Deserticolin-1 and then used to infect cells; Cell pre-treatment: Hylaseptin-P1 and Deserticolin-1 incubated with the cells before the viral infection; Post-treatment: Hylaseptin-P1 and Deserticolin-1 added to the infected cells. Infected cells were used as negative control (ctr-). **** $p < 0.0001$; ns: non-significant.

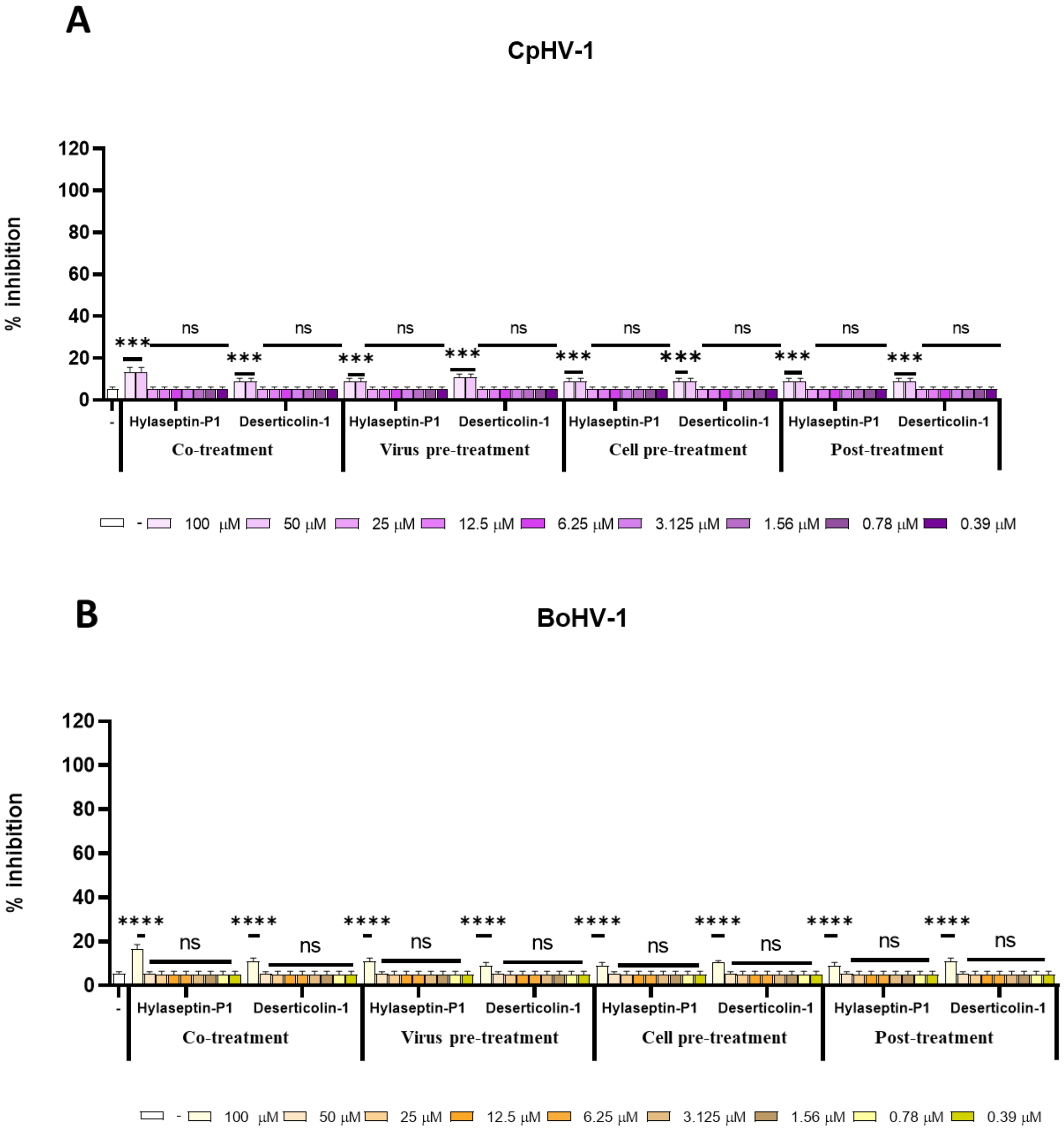

Figure S4. Antiviral activity against CpHV-1 and BoHV-1. Different assays were performed to evaluate anti-CpHV-1 (**A**) and anti-BoHV-1 (**B**) activities. From left to right: Co-treatment: simultaneous addition of peptide and virus to the cells; Virus pre-treatment: virus incubated with Hylaseptin-P1 and Deserticolin-1 and then used to infect cells; Cell pre-treatment: Hylaseptin-P1 and Deserticolin-1 incubated with the cells before the viral infection; Post-treatment: Hylaseptin-P1 and Deserticolin-1 added to the infected cells. Infected cells were used as negative control (ctr-). **** $p < 0.0001$; *** $p = 0,0003$; ns: non-significant.
